# 

**Published:** 1874-06

**Authors:** 


### Contents of June Number.

### ORIGINAL DEPARTMENT.

ART. I—The Nervous Diseases of Women. By Allan McLane Hamilton, M. D .... 401

ART. II—Recent Investigations in the Physiological Functions of the Brain. By H. R. Bigelow, M. D.... 405

ART. III—Albany County Medical Society. Semi-Monthly Meeting, March 25th. Reported by F. C Curtis, M. D., Secretary.... 415

ART. IV—Abstract of Proceedings of Buffalo Medical Association, May, 1874. Reported by E. R. Barnes, M. D , Secretary.... 426

### EDITORIAL DEPARTMENT.

American Medical Association.... 433

Passage of the Bill to regulate the rank of the Staff of the U. S. Army. 436

A Law regulating the Practice of Medicine and Surgery.... 436

Prof. F. H. Hamilton, M. D., and the N. Y. City Commissioners of Public Charities and Correction.... 437

Books and Pamphlets Received.... 440

